# *Yersinia pseudotuberculosis* and *Y. enterocolitica* abortions in sheep and goats in California: a series of cases diagnosed at CAHFS laboratories, 2002–2023

**DOI:** 10.1177/10406387251324883

**Published:** 2025-03-11

**Authors:** Seung-Hee Cho, Aslı Mete, Isaiah Cueva, Melissa Macías-Rioseco, Heather Fritz, Nicolas Streitenberger, Omar Gonzales-Viera

**Affiliations:** Department of Comparative Pathobiology, College of Veterinary Medicine, Purdue University, West Lafayette, IN, USA; California Animal Health and Food Safety Laboratory, Davis, branches, University of California–Davis, Davis, CA, USA; California Animal Health and Food Safety Laboratory, Davis, branches, University of California–Davis, Davis, CA, USA; Tulare, branches, University of California–Davis, Davis, CA, USA; California Animal Health and Food Safety Laboratory, Davis, branches, University of California–Davis, Davis, CA, USA; California Animal Health and Food Safety Laboratory, Davis, branches, University of California–Davis, Davis, CA, USA; California Animal Health and Food Safety Laboratory, Davis, branches, University of California–Davis, Davis, CA, USA

**Keywords:** abortion, California, epidemiology, pathology, small ruminants, yersiniosis

## Abstract

Abortion in small ruminants poses a significant economic threat and can have zoonotic risk. Although the association between yersiniosis and reproductive complications is known, systematic studies and case series on abortion in sheep and goats are scarce. Here we describe epidemiologic and pathologic findings in 34 cases of *Yersinia pseudotuberculosis*– and *Y. enterocolitica*–associated abortions in sheep and goats, contributing to the understanding of these zoonotic diseases in California. We conducted a 22-y retrospective study to examine microbiologic and pathologic findings in abortion submissions, as well as the geographic and seasonal distribution of the analyzed cases. Yersiniosis-induced abortion was diagnosed in 22 goats and 12 sheep, with all abortions occurring in the last third of gestation. Samples from lung, liver, placenta, and abomasal contents were submitted for aerobic culture; the highest recovery of *Yersinia* spp. was from abomasal contents. Microscopically, there was severe necrotizing and suppurative inflammation in the lung, liver, spleen, kidney, and, when present, the placenta, with intralesional bacterial colonies. All cases were received from northern and central California in the winter and spring. Our study provides epidemiologic and pathologic features of *Yersinia* spp.–induced abortions in small ruminants and provides critical data to pave the way for future research, surveillance, and preventive strategies.

Abortion in small ruminants can be caused by various non-infectious^16^ and infectious agents^4,15,16,18,23^ and leads to enormous economic losses and zoonotic diseases.^3,12^ Because both *Yersinia pseudotuberculosis* and *Y. enterocolitica* can cause infections in humans through ingestion of the organisms, they are among the pathogens that must be considered as potential zoonotic agents associated with small ruminant abortions.^12^ Among the infectious etiologies, information on *Yersinia* spp. causing ovine and caprine abortions is limited.^7,8,24,25^

*Y. pseudotuberculosis* is a gram-negative coccobacillus that shares the genus with *Y. enterocolitica*.^17^ Optimal growth conditions for *Yersinia* are <28–29°C on culture and in cold weather.^11,20^ *Y. pseudotuberculosis* is known to cause enterotyphlocolitis, mastitis, septicemia, lymphadenitis, abortion, neonatal death, and ocular disease in goats.^14^ Infection of ewes with *Y. pseudotuberculosis*, while not reported commonly, can lead to placentitis, abortion, stillbirth, or birth of weak or healthy lambs.^8,9,15,17^

*Y. enterocolitica* is a gram-negative bacillus and has been isolated from aborted lambs.^5,24^ An experimental study confirmed the abortigenic potential of *Y. enterocolitica* O serotype in ewes, resulting in placentitis and abortion, with subsequent normal pregnancies.^6^

In California, a retrospective study of *Y. pseudotuberculosis*–associated disease included 42 goats from 1990 to 2012 (23 y) and found a strong seasonal pattern in winter and spring.^14^ The most frequent lesions were enteritis and/or typhlocolitis, followed by abscessation, abortion, conjunctivitis, and hepatitis.^14^ Abortion occurred in 5 of 42 (12%) goat cases.^14^

However, most cases of yersiniosis-induced abortions in small ruminants are of individual animals or small groups, and no case series in sheep and goats have been published, to our knowledge. Therefore, our objective was to describe the epidemiologic and pathologic features of the abortions caused by *Y. pseudotuberculosis* and *Y. enterocolitica* in goats and sheep, submitted to the California Animal Health and Food Safety Laboratory System (CAHFS) over 22 y, aiming to contribute to the knowledge of this zoonotic disease in California.

## Materials and methods

We searched the Laboratory Information Management System (LIMS), across all 4 branches of the CAHFS (Davis, Turlock, Tulare, and San Bernardino, CA, USA), using the terms “Yersin” and “abortion” in the diagnosis field for ovine and caprine submissions received between May 23, 2002, and December 31, 2023 (22 y). The inclusion criteria were all abortion cases in sheep and goats, in which either *Y. pseudotuberculosis* or *Y. enterocolitica* was isolated and considered the primary pathogen, defined as the dominant pathogen cultured or detected associated with the macroscopic and/or microscopic lesions. Infectious abortifacients of small ruminants, including *Brucella*, *Campylobacter* spp., bovine viral diarrhea/border disease viruses (BVDV, BDV), and *Leptospira* spp., were ruled out by culture (*Brucella*, *Campylobacter* spp.) or PCR testing (BVDV, BDV, *Leptospira* spp.).

The “Summary” and “Diagnosis fields” data on each final report were analyzed case by case. Sex, fetal crown-rump length (CRL), and fetal gestational age were retrieved from the dataset. CRLs were used to estimate the fetal age when the age was not recorded in the report.^22^ Gross and histologic lesions were also recorded. Concurrent bacterial infections recovered from the lung, liver, spleen, and placenta were also considered. Bacteriologic techniques used included both routine aerobic culture and *Yersinia* cold-enrichment cultures.^14^ For routine aerobic culture, tissues were sampled with cotton-tipped swabs that were inoculated onto 5% sheep blood agar (SBA) and MacConkey agar (MAC), streaked for isolation, and incubated at 35 ± 2°C with 5–10% CO_2_ for 24–48 h. For *Yersinia* cold enrichment, a tissue swab was transferred into PBS and stored at 4°C for 3 wk. Once a week, the cold-enrichment broth was subcultured onto MAC and cefsulodin–irgasan–novobiocin agar (CIN) and incubated at 23–26°C for 42–48 h, with evaluation for growth at 18–24 and 42–48 h. Bacterial isolates were identified using a combination of biochemical testing and matrix-assisted laser desorption/ionization time-of-flight mass spectrometry (MALDI-TOF MS; Bruker).

Heavy metals (copper, zinc, lead, manganese, iron, mercury, arsenic, molybdenum, cadmium) and selenium were quantified in the liver tissues,^13^ if the pathologist requested the analysis. The date when the carcasses were submitted to the laboratory (temporal distribution) was classified as the seasonal section; December, January, and February were considered winter, and March, April, and May were considered spring. The animals’ geographic location was also retrieved and mapped based on the county of the collection site.

## Results

A total of 1,106 abortion submissions (746 caprine, 360 ovine) were submitted to the CAHFS during our retrospective study time, excluding submissions from out of state; 17 submissions (1.5%; 11 caprine, 6 ovine) were diagnosed as yersiniosis-associated abortions. These 17 submissions comprised 34 small ruminant aborted fetuses (22 caprine, 12 ovine cases), which were analyzed separately (Suppl. Table 1). From the caprine cases, 3 submissions consisted of 2 cases each, including 2 sets of twins; 4 submissions comprised 3 cases each. In sheep, 4 submissions featured 2 cases each, and 1 submission included 3 cases.

Among the 22 caprine cases, 10 were males with CRLs of 29–43 cm. Nine fetuses were females with CRLs of 29–42 cm. Among 12 ovine specimens, the males (6 of 12) had CRLs of 32–50 cm; the female (4 of 12) CRLs were 35–39 cm. The CRL was not recorded in 3 goat and 2 sheep fetuses. Based on the submission forms and CRLs,^22^ all abortion cases occurred during the last third of gestation.

**Figure 1.**
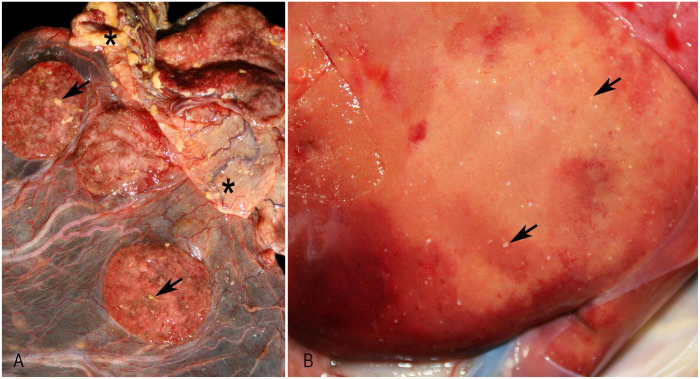
Gross findings of *Yersinia pseudotuberculosis* abortion. **A.** Purulent exudate present on the surface of intercotyledonary areas (asterisks) and over the cotyledons (arrows) of the placenta in a goat. **B.** Myriad necrotic foci throughout the hepatic parenchyma (arrows) in a sheep.

In 34 cases, 23 fetuses had unremarkable gross findings. Among 11 cases, random pinpoint pale-tan necrotic foci were found in 8 livers, 3 spleens, 2 lungs, 2 kidneys, and 1 placenta (Fig. 1), and hemorrhage was found in 1 placenta.

**Figure 2.**
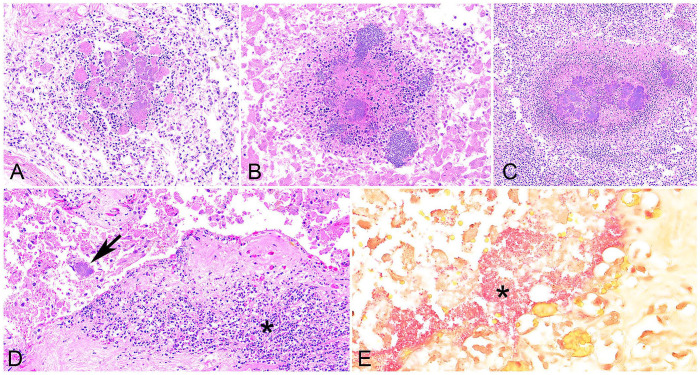
Histopathologic features of yersiniosis-induced abortion in goats. **A.** Necrotizing pneumonia. **B.** Hepatitis. **C.** Splenitis. The lesions efface the lung, hepatic, and splenic parenchyma multifocally, and have prominent central eosinophilic necrotic foci with bacterial colonies (bacilli) surrounded by dense degenerate nuclei and light eosinophilic necrotic parenchyma. H&E. **D.** Necrosuppurative placentitis. Dense inflammatory infiltrates within the decidual plate (asterisk), and basophilic bacterial colonies are visible (arrow). H&E. **E.** Gram-negative, pink coccobacilli (asterisk) within the placenta of Fig. 2D. Gram stain.

Four types of histologic lesions were mainly observed. Necrotizing inflammation was described when necrotic tissue with degenerate leukocytes was dominant. Viable neutrophils predominated in suppurative inflammation. Necrosuppurative inflammation referred to necrotic foci surrounded by degenerate and viable neutrophils. Pleocellular inflammation was used when there was a mixed population of inflammatory cells without a dominant cell type. Placentitis was the most frequent histologic lesion, occurring in 14 of 16 (88%) cases. The following major organs with inflammation were the lung, liver, spleen, and kidney (Table 1; Fig. 2). Intralesional colonies of coccobacilli were observed in 10 placentas, 11 lungs, 15 livers, 10 spleens, and 6 kidneys.

**Table 1.** Frequency of histopathologic findings in 34 *Yersinia* spp. aborted fetuses in goats and sheep.

	Histologic findings				Lesions/Total examined tissues
Condition	Necrotizing	Suppurative	Necrosuppurative	Pleocellular	
Placentitis	3	6	3	2	14/16 (88%)
Pneumonia	6	11	6	4	27/34 (79%)
Hepatitis	14	1	2	3	20/34 (59%)
Splenitis	12	0	2	0	14/34 (41%)
Nephritis	6	0	2	0	8/34 (24%)

The histologic findings were indistinguishable in cases of *Y. pseudotuberculosis* from goats and sheep. Among *Y. enterocolitica*–positive caprine cases, 3 fetuses from the same submission had suppurative pneumonia and pleocellular hepatitis. One placenta from this submission had necrotizing lesions. Another *Y. enterocolitica*–positive fetus did not have any inflammatory or necrotizing lesions.

Aerobic cultures were done from 30 lung swabs, 29 liver swabs, 24 abomasal contents, and 15 placental swabs for *Y. pseudotuberculosis*, and from 4 lung swabs, 4 liver swabs, 4 abomasal contents, and 1 placental swab for *Y. enterocolitica* (Table 2). *Y. pseudotuberculosis* was most frequently isolated from abomasal contents, followed by lung, liver, and placental swabs.

**Table 2.** Result of bacterial aerobic culture in cases of *Yersinia* spp. abortion in goats and sheep.

Aerobic culture	Lung	Liver	Abomasal contents	Placenta
*Y. pseudotuberculosis*	27/30 (90%)	25/29 (86%)	23/24 (96%)	10/15 (67%)
*Y. enterocolitica*	3/4 (75%)	3/4 (75%)	2/4 (50%)	1/1 (100%)

The denominator is the number of tests in which aerobic culture was performed, and the numerator is the number of positive results for that test.

Metal quantification was conducted in the liver of 7 caprine fetuses (3 submissions), per the case coordinator. Among them, selenium quantification was conducted in the liver of 4 caprine fetuses (2 submission). Notably, these tests detected deficiencies of copper, zinc, manganese (7 cases each), and selenium (4 cases).

**Figure 3.**
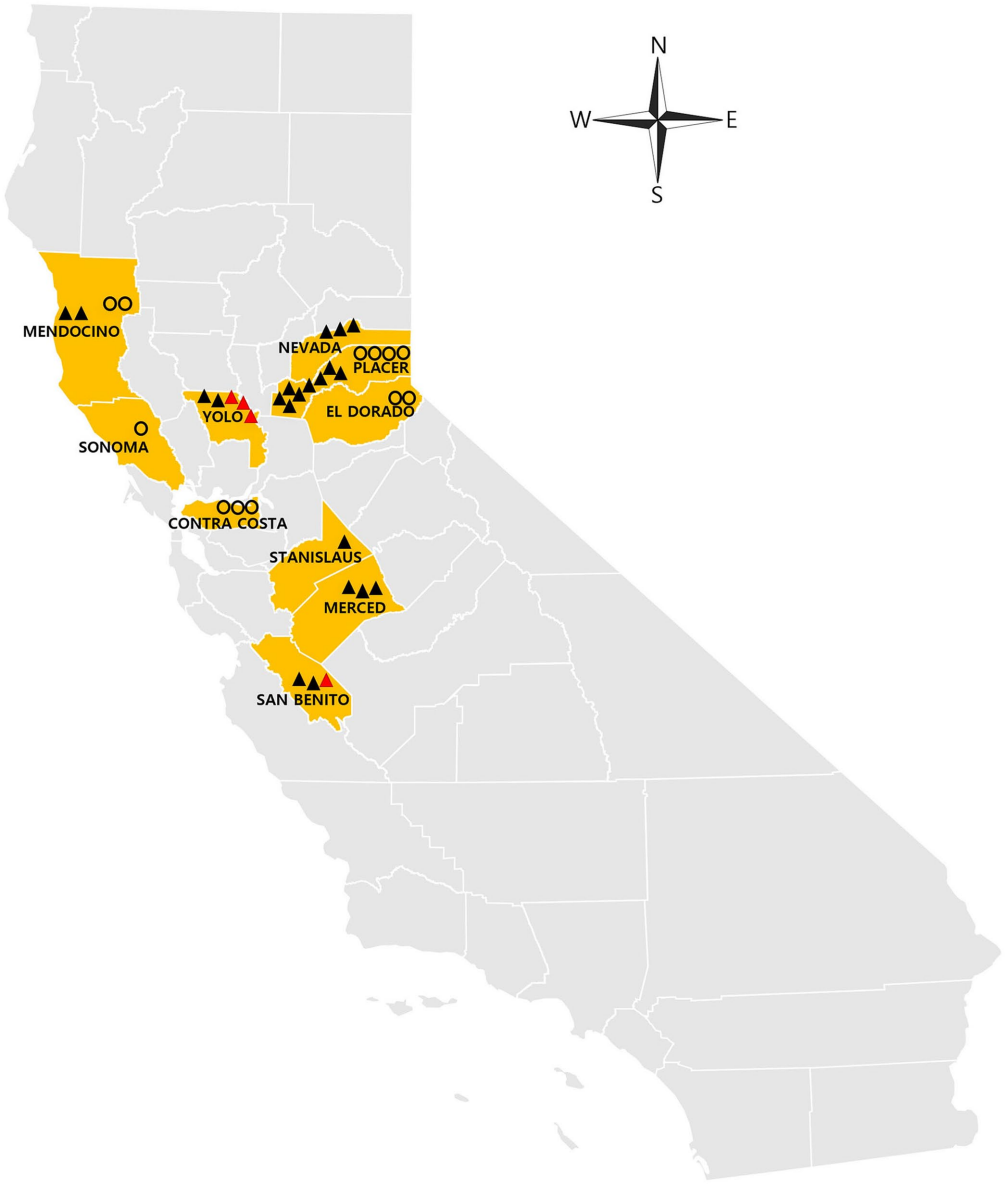
Geographic distribution of 34 cases of *Yersinia pseudotuberculosis* and *Y. enterocolitica* abortion in goats and sheep in California. Black triangles represent caprine abortion cases caused by *Y. pseudotuberculosis*, red triangles represent caprine abortion cases caused by *Y. enterocolitica*, and circles represent ovine abortion cases caused by *Y. pseudotuberculosis*.

Among the 1,106 total abortion submissions to CAHFS from 2002 to 2023, 780 were submitted to the Davis branch, 219 to the Tulare branch, and 107 to the San Bernardino branch. Submission numbers of yersiniosis abortion were comprised of 13 *Y. pseudotuberculosis* and 2 *Y. enterocolitica* cases at the Davis branch, and 2 *Y. pseudotuberculosis* cases at the Tulare branch. Among the counties represented, 7 were located in northern California (Contra Costa, El Dorado, Mendocino, Nevada, Placer, Sonoma, Yolo counties), and 3 counties were in central California (Merced, San Benito, Stanislaus counties; Fig. 3). Among *Y. pseudotuberculosis* abortion cases, caprine cases were distributed in both the northern and central parts of California, whereas ovine cases were mainly located in northern California. Caprine *Y. enterocolitica* abortion cases originated from Yolo County (3 cases) and San Benito County (1 case). None of the caprine or ovine *Yersinia* abortions were from animals located in southern California.

The seasonal distribution of the 17 submissions of yersiniosis small ruminant abortion revealed a spring and winter pattern (Table 3). In spring, 8 submissions were recorded, including 5 from goats and 3 from sheep (8 of 319 total submissions; 2.5%). In winter, 9 submissions were reported, comprising 6 from goats and 3 from sheep (9 of 557 total submissions; 1.6%). No yersiniosis abortions were submitted during summer or autumn.

**Table 3.** Seasonal distribution of 17 submissions of *Yersinia* spp. abortion in goats and sheep from California.

	*Yersinia* spp. abortion/total small ruminant abortion				
Species	Spring	Summer	Autumn	Winter	Total
Goat	5/259	0/51	0/84	6/352	11/746
Sheep	3/60	0/10	0/85	3/205	6/360
Total	8/319	0/61	0/169	9/557	17/1,106

## Discussion

Our comprehensive analysis of yersiniosis-induced abortion cases in small ruminants received at the CAHFS laboratories unveiled crucial insights into the epidemiologic and pathologic aspects of these infections. A 2001 retrospective study of 211 goat abortion cases in California^19^ and a 2022 retrospective study of 100 sheep abortion cases in Uruguay^9^ did not report any *Yersinia* spp.–associated abortions, suggesting that yersiniosis is an uncommon cause of abortion in small ruminants.

Among the *Yersinia*-associated abortions in our study, *Y. pseudotuberculosis* was the predominant etiologic agent. The pathologic findings, such as necrotizing and/or suppurative inflammation in multiple organs, were similar to those described in previous reports.^14,17^

Consistent with prior reports,^5,21,24^ *Y. enterocolitica* was isolated from both caprine and ovine abortion cases in our study. Fetal histopathologic features of the goat abortions were similar to those of the previous studies in sheep, such as systemic suppurative and necrotizing inflammation.^5,6^ These findings indicate that in addition to *Y. pseudotuberculosis*, *Y. enterocolitica* also causes caprine and ovine abortions in California. Experimental studies would provide more details about the pathogenesis of *Y. enterocolitica* as an abortigenic pathogen in goats. The aerobic culture method using *Yersinia* selective agars could not reveal serotypes or more detailed information about the pathogen. Serotyping would be needed to confirm whether serotype O, from the previously diagnosed ovine abortions,^6^ caused the goat losses.

Among the specimens examined histologically, the placenta was more frequently affected by inflammation, followed by the lung, liver, spleen, and kidney. These findings suggest the infection sequence, commencing from maternal infection, of progression through placental involvement, followed by aspiration into the lungs and subsequent spread to other organs. This hierarchical frequency pattern of inflammation underscores the propensity for organs infected earlier to manifest higher positivity frequency. Moreover, this observation is correlated with the high frequency of positive results observed in abomasal contents via aerobic bacterial culture, implying that fetuses swallow infected amniotic fluid.^10^

In the *Y. pseudotuberculosis* cases, there were no differences in the type of lesions in ovine or caprine cases. Necrosis with suppurative and/or pleocellular inflammation was a noticeable feature in the liver, spleen, and kidney. This result is consistent with a study that reported necrotizing and/or suppurative lesions in multiple fetal organs.^14^ Suppurative inflammation was the most frequent inflammatory subtype in the placenta and lung.

*Y. enterocolitica* was positive in only 4 caprine cases; therefore, there were limitations in analyzing the pattern of lesions. However, among *Y. enterocolitica–*positive caprine cases, 3 fetuses from the same submission had suppurative pneumonia and hepatitis, and 1 fetus had necrotizing placentitis. These histopathologic findings are similar to those observed in *Y. pseudotuberculosis* abortion cases. The last *Y. enterocolitica*–positive fetus had no inflammatory or necrotizing lesion.

Many of the tissues examined microscopically had dense colonies of intralesional bacteria in several cases, suggesting these as true antemortem changes. However, in cases in which the bacteria did not form dense colonies, considering that autolysis/postmortem decomposition is often advanced in fetuses at the time of diagnosis, we cannot rule out that the observed bacteria, even if found histologically alongside inflammation, are not *Yersinia* spp.

Aerobic cultures confirmed the presence of *Y. pseudotuberculosis* and *Y. enterocolitica* in various sample types. Abomasal contents were most frequently culture-positive, suggesting that abomasal contents are the crucial sample to test for *Yersinia* spp. However, there was variation in the frequency of positive results across the different tissues tested, emphasizing the importance of sampling multiple affected tissues and correlating aerobic culture results with histologic findings, such as the presence of intralesional gram-negative coccobacilli often forming dense colonies. Several cases had other concurrent bacterial recoveries, such as *Escherichia coli*, detected in small or rare quantities from aerobic culture. It was hypothesized that these bacteria might represent opportunistic infections or postmortem contaminants or overgrowth. Given that *Yersinia* spp. are not typically considered opportunistic pathogens, and other pathogens were infrequently isolated in significant numbers compared to *Yersinia* spp., the focus of the diagnosis was directed towards yersiniosis abortion. Nonetheless, these coinfections may have contributed to lesions that deviated from the typical manifestations of yersiniosis.

In livestock, copper and selenium deficiencies have been associated with decreased resistance to bacterial infections.^1,14^ We identified hepatic copper, zinc, and magnesium deficiency in 7 of 34 cases and selenium deficiency in 4 of those 7 cases, suggesting the possible involvement of these deficiencies in the pathogenesis of *Yersinia* spp. abortions. This possible link between nutritional deficiencies and bacterial infections may offer actionable insights for improved herd management practices to mitigate reproductive losses in small ruminants.

Geographic distribution analysis revealed a cluster of cases in northern and central California. For seasonal distribution, in California, the natural parturition period for sheep and goats occurs in the spring,^2,7^ and in our study, CAHFS received more small ruminant abortions during spring (319 submissions) and winter (557 submissions) compared to summer (61 submissions) and autumn (169 submissions). Thus, the concentration of *Yersinia* abortions, in northern and central California with colder climates during the winter and spring seasons, may be related to the higher number of abortion submissions, corresponding to their populations and seasonal breeding. However, this may suggest a plausible association with the well-known preference of *Yersinia* for more frigid environments.^11,20^ Understanding regional variations and seasonal influences on *Yersinia* prevalence is crucial for targeted surveillance and preventive measures.

## Supplemental Material

sj-pdf-1-vdi-10.1177_10406387251324883 – Supplemental material for Yersinia pseudotuberculosis and Y. enterocolitica abortions in sheep and goats in California: a series of cases diagnosed at CAHFS laboratories, 2002–2023 Supplemental material, sj-pdf-1-vdi-10.1177_10406387251324883 for Yersinia pseudotuberculosis and Y. enterocolitica abortions in sheep and goats in California: a series of cases diagnosed at CAHFS laboratories, 2002–2023 by Seung-Hee Cho, Aslı Mete, Isaiah Cueva, Melissa Macías-Rioseco, Heather Fritz, Nicolas Streitenberger and Omar Gonzales-Viera in Journal of Veterinary Diagnostic Investigation
