# Supplementary material for: Yersinia pseudotuberculosis and Y. enterocolitica abortions in sheep and goats in California: a series of cases diagnosed at CAHFS laboratories, 2002–2023
Source: J Vet Diagn Invest. 2025 Mar 11;37(4):545–51. doi: 10.1177/10406387251324883 (PMC11904924; doi:10.1177/10406387251324883)
Supplement: sj-pdf-1-vdi-10.1177_10406387251324883 – Supplemental material for Yersinia pseudotuberculosis and Y. enterocolitica abortions in sheep and goats in California: a series of cases diagnosed at CAHFS laboratories, 2002–2023 [file sj-pdf-1-vdi-10.1177_10406387251324883.pdf]

Cho S-H, et al. *Yersinia pseudotuberculosis* and *Y. enterocolitica* abortions in sheep and goats in California: a series of cases diagnosed at CAHFS laboratories in 2002–2023

Supplemental Table 1. *Yersinia pseudotuberculosis* and *Y. enterocolitica* abortions in sheep and goats in California: cases diagnosed at the CAHFS laboratory in 2002–2023.

			Histology					Bacteriology				Toxicology
ID	Species	Season	Lung	Liver	Placenta	Spleen	Kidney	Lung	Liver	Placenta	Abomasal contents	Deficiency
1	Caprine	Spring	PP	NH, IB	—	NS	NN, IB	Y. pseudo	Y. pseudo	Y. pseudo	Y. pseudo	NA
2	Caprine	Spring	—	—	—	—	—	Mixed	Mixed	NA	Y. entero	NA
3	Caprine	Winter	NP, IB	NH, IB	—	NS, IB	NN, IB	Y. pseudo	Y. pseudo	NA	Y. pseudo	NA
4	Caprine	Winter	NP, IB	NH, IB	—	NS, IB	NN, IB	Y. pseudo	Y. pseudo	NA	Y. pseudo	NA
5	Caprine	Winter	NP, IB	—	—	—	—	Y. pseudo	Y. pseudo	NA	Y. pseudo	NA
6	Caprine	Winter	SP	SH	NSPl, IB	NS	—	Y. pseudo	Y. pseudo	*E. coli*	NA	NA
7	Caprine	Winter	—	—	—	—	—	Y. pseudo	No growth	NA	NA	NA
8	Caprine	Winter	SP	—	—	—	—	Y. pseudo	No growth	NA	NA	NA
9	Caprine	Spring	NP, IB	NH, IB	NSPl, IB	NS, IB	NN, IB	Y. pseudo	Y. pseudo	Y. pseudo	Y. pseudo	NA
10	Caprine	Spring	SP	—	—	—	—	Y. pseudo	Y. pseudo	NA	Y. pseudo	NA
11	Caprine	Winter	PP	—	NPl, IB	—	—	No growth	*E. coli*	*E. coli*	Y. pseudo	NA
12	Caprine	Winter	—	—	NPl, IB	—	—	Y. pseudo	Y. pseudo	*E. coli*	Y. pseudo	NA
13	Caprine	Winter	—	—	—	—	—	Y. pseudo	Y. pseudo	NA	Y. pseudo	NA
14	Caprine	Spring	SP	PH	NPl	—	—	Y. entero	Y. entero	Y. entero	No growth	NA
15	Caprine	Spring	SP	PH	—	—	—	Y. entero	Y. entero	Y. entero	No growth	NA
16	Caprine	Spring	SP	PH	—	—	—	Y. entero	Y. entero	NA	Y. entero	NA
17	Caprine	Winter	PP, IB	NH, IB	SPl, IB	NS, IB	NN, IB	Y. pseudo	Y. pseudo	Y. pseudo	Y. pseudo	NA
18	Caprine	Winter	SP	—	SPl, IB	—	—	Y. pseudo	Y. pseudo	Y. pseudo	Y. pseudo	Copper, zinc, manganese, selenium

19	Caprine (twin)	Winter	—	—	—	—	—	Mixed	Y. pseudo	*E. coli*	No growth	Copper, zinc, manganese, selenium
20	Caprine (twin)	Winter	NP, IB	NH, IB	—	NS, IB	NN, IB	Y. pseudo	Y. pseudo	NA	Y. pseudo	Copper, zinc, manganese, selenium
21	Caprine (twin)	Winter	NP	NH, IB	—	NS, IB	NSN	Y. pseudo	Y. pseudo	NA	Y. pseudo	NA
22	Caprine (twin)	Winter	NSP	NH, IB	—	NS, IB	NSN	Y. pseudo	Y. pseudo	NA	Y. pseudo	Copper, zinc, manganese, selenium
23	Ovine	Winter	NSP, IB	NH, IB	SPl, IB	NS, IB	—	Y. pseudo	Y. pseudo	Y. pseudo	Y. pseudo	Copper, zinc, manganese
24	Ovine	Winter	NSP, IB	NH, IB	SPl, IB	—	—	Y. pseudo	Y. pseudo	Y. pseudo	NA	Copper, zinc, manganese
25	Ovine	Winter	NP	NH	PPl, IB	—	—	No growth	No growth	*E. coli, Acinetobacter* sp.	Y. pseudo	Copper, zinc, manganese
26	Ovine	Winter	NP	NH, IB	—	—	—	Y. pseudo	Y. pseudo	NA	Y. pseudo	NA
27	Ovine	Winter	—	—	SPl, IB	—	—	Y. pseudo	Y. pseudo	Y. pseudo	Y. pseudo	NA
28	Ovine	Winter	—	—	—	—	—	Y. pseudo	Y. pseudo	NA	Y. pseudo	NA
29	Ovine	Winter	PP, IB	—	—	—	—	Y. pseudo	NA	NA	Y. pseudo	NA
30	Ovine	Winter	NSP, IB	NSH, IB	—	NSS	—	Y. pseudo	Y. pseudo	NA	NA	NA
31	Ovine	Winter	NSP, IB	NSH, IB	SPl	NSS	—	Y. pseudo	Y. pseudo	Y. pseudo	NA	NA
32	Ovine	Spring	SP	NH, IB	NSPl	NS, IB	—	Y. pseudo	Y. pseudo	Y. pseudo	Y. pseudo	NA
33	Ovine	Spring	SP	NH, IB	PPl	NS, IB	—	Y. pseudo	Y. pseudo	Y. pseudo	Y. pseudo	NA
34	Ovine	Spring	SP	—	—	—	—	Y. pseudo	Y. pseudo	NA	Y. pseudo	NA

— = no significant findings; IB = intralesional bacteria; NA = not applicable; NH = necrotizing hepatitis; NN = necrotizing nephritis; NP = necrotizing pneumonia; NPl = necrotizing placentitis; NS = necrotizing splenitis; NSH = necrosuppurative hepatitis; NSN = necrosuppurative nephritis; NSP = necrosuppurative pneumonia; NSPl = necrosuppurative placentitis; NSS = necrosuppurative

splenitis; PH = pleocellular hepatitis; PP = pleomorphic pneumonia; PPl = pleocellular placentitis; SH = suppurative hepatitis; SP = suppurative pneumonia; SPl = suppurative placentitis; Y. entero = *Yersinia enterocolitica*; Y. pseudo = *Yersinia pseudotuberculosis.*
